# Sex-Specific Differences in the Gut Microbiome in Response to Dietary Fiber Supplementation in IL-10-Deficient Mice

**DOI:** 10.3390/nu12072088

**Published:** 2020-07-15

**Authors:** Zhengxiao Zhang, Jae Eun Hyun, Aducio Thiesen, Heekuk Park, Naomi Hotte, Hikaru Watanabe, Takanobu Higashiyama, Karen L. Madsen

**Affiliations:** 1 Department of Medicine, University of Alberta, Edmonton, AB T6G 2E1, Canada; zh16@ualberta.ca (Z.Z.); jhyun2@ualberta.ca (J.E.H.); athiesen@ualberta.ca (A.T.); nhotte@ualberta.ca (N.H.); 2 The Centre of Excellence for Gastrointestinal Inflammation and Immunity Research (CEGIIR), University of Alberta, Edmonton, AB T6G 2E1, Canada; 3 Department of Medicine, Columbia University, New York, NY 10031, USA; hp2523@cumc.columbia.edu; 4 Hayashibara Co., Ltd., Okayama 700-0041, Japan; hikaru.watanabe@hb.nagase.co.jp (H.W.); takanobu.higashiyama@hb.nagase.co.jp (T.H.)

**Keywords:** sex differences, gut microbiota, dietary fiber, isomaltodextrin, colitis, cytokines

## Abstract

There is growing interest in studying dietary fiber to stimulate microbiome changes that might prevent or alleviate inflammatory bowel disease (IBD). However, dietary fiber effects have shown varying degrees of efficacy, for reasons that are unclear. This study examined whether the effects of isomaltodextrin on gut microbiota and IBD were dependent on dose or host sex, using an Interleukin (IL)-10 deficient murine colitis model. After 12 weeks, colonic IL-12p70 was depressed in male mice receiving high-dose isomaltodextrin supplementation compared to the control group (*p* = 0.04). Male mice receiving high-dose isomaltodextrin exhibited changes in microbial alpha-diversity, including enhanced richness and evenness (*p* = 0.01) and limited reduction in the relative abundance of *Coprococcus* (*q* = 0.08), compared to the control group. These microbial compositional changes were negatively associated with IL-12p70 levels in the male group (rs ≤ −0.51, *q* ≤ 0.08). In contrast, female mice receiving isomaltodextrin displayed a reduction in alpha-diversity and *Coprococcus* abundance and a high level of IL-12p70, as did the control group. Together, these results indicate that isomaltodextrin altered the gut microbial composition linking specific immune-regulatory cytokine responses, while the interactions among fiber, microbiota and immune response were dose dependent and largely sex specific. The results further indicate that interactions between environmental and host factors can affect microbiome manipulation in the host.

## 1. Introduction

Inflammatory bowel diseases (IBD), including Crohn’s disease (CD) and ulcerative colitis (UC), are complex, chronic, immune-mediated inflammatory disorders of the gastrointestinal tract [1]. IBD is most prevalent in developed countries, especially in Europe and North America [2]. It has been hypothesized that increased consumption of processed foods together with a reduced intake of dietary fiber may contribute to the onset of disease and increase severity [3,4]. Increasing evidence has implicated host immune–microbial interactions and luminal microbial dysbiosis in the pathogenesis of IBD [5,6]. Several studies have reported a reduction in microbial species within the phylum Firmicutes and the genus *Faecalibacterium* and *Roseburia*, known producers of short chain fatty acids (SCFAs) [7,8,9,10]. SCFAs are a carbon energy source for intestinal epithelial cells and favor induction of regulatory T (Treg) cells with potent anti-inflammatory functions [11,12]. Although the cause of IBD is unknown, targeting of host–microbial interactions and supplementation of SCFAs or stimulating the growth of the gut microbes that produce them may benefit patients with IBD.

Dietary fiber refers to a heterogeneous group of plant-derived carbohydrates, which are non-digestible by the host and selectively fermented by gut microbes [13]. In animal models, high fiber intake can alter the gut microbiota and reduce inflammation along with enhancing gut barrier function [14,15,16]. However, human randomized control trials using dietary fiber in the treatment of IBD have shown varying degrees of efficacy and the reasons are unclear [17,18]. One possible reason is that the response of individuals to fiber supplementation is dependent upon the structure of their unique microbial community, which can be highly variable with regards to microbial shifts and metabolite production [19]. The individual variation in the structure of the gut microbiota community is determined by environmental and host factors [20,21]. In addition, while it is uncertain if biological sex differences have an impact on the composition of gut microbiota, animal studies have demonstrated that immune and hormonal variations based on the host’s sex can impact the composition and metabolism of gut bacterial communities [22,23]. Interestingly, a cross-sectional study suggested that the sex of the subject affects the relationship between dietary fiber intake and the gut microbiota composition [24]. Together, these findings lead to the hypothesis that sex differences may influence the interactions among fiber, microbiota and immune response. However, few studies have investigated the sex-dependent effects of dietary fiber supplementation in IBD. In addition, from a practical perspective, the necessary fiber dosage required for inducing reproducible changes in the microbiome is not well documented [25]. A recent study showed a dose dependency of dietary fiber-induced effects on the specific bacterial composition [26], which encourages exploring whether necessary dose-dependency of fiber also affects the host immuno-metabolic response. 

In this context, our study examined the dose-dependent effects of dietary fiber on immune cytokine production, histopathology and the gut microbiota in an Interleukin 10 (IL-10) knock out (IL-10^−/−^) murine colitis model. The soluble dietary fiber that was tested in this study was a highly branched α-glucan, isomaltodextrin (IMD). IMD is a soluble dietary fiber produced from starch using *α-glucosyltransferase* and *α-amylase* derived from *Paenibacillus alginolyticus* PP710 [27]. Previous rodent studies demonstrated anti-inflammatory effects of IMD in mice in a dextran sulfate sodium (DSS)-induced colitis model [28] and in a lipopolysaccharide-induced low-grade chronic inflammation model [29]. In this study, we aimed to assess the effect of IMD on colitis development and gut microbiota, and to further explore if the response was sex-specific in a genetic model of colitis.

## 2. Materials and Methods

### 2.1. Animal Experiment

**Figure 1:**
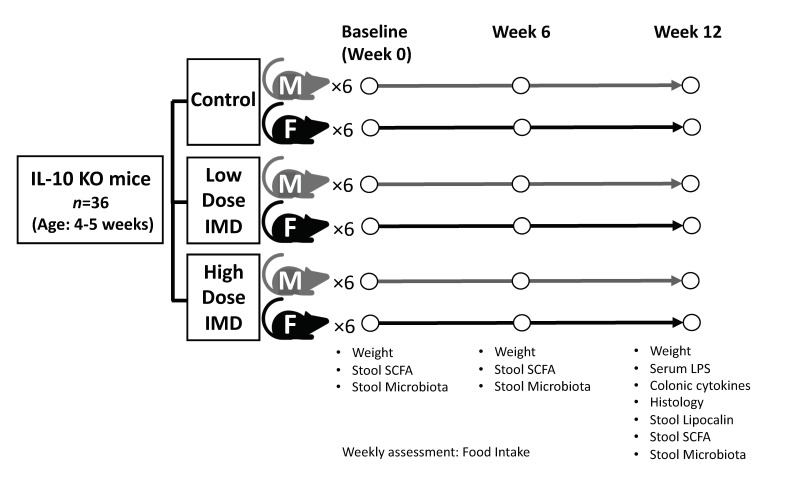
Experimental design. Abbreviation: IL, interleukin; KO, Knockout; IMD, Isomaltodextrin; M, Male; F, Female; SCFA, Short chain fatty acid; LPS, Lipopolysaccharide

IL-10 deficient 129(B6)- IL-10^tm1Cgn^/J mice were bred at the Health Sciences Laboratory Animal Services at the University of Alberta (Edmonton, Canada). Protocols were approved by the animal care committee at the University of Alberta. This study was performed in three separate cohorts from different litters over a period of six months. All mice were kept under 25 °C and 45–55% humidity. The study design is shown in Figure 1. At four–five weeks of age, IL-10^−/−^ mice (*n* = 36) were randomly assigned to three different dietary groups: control diet with cellulose, low dose isomaltodextrin (IMD) diet, or high dose IMD diet with males (*n* = 6) and females (*n* = 6) in each group. Previous reports found 5% to be a high-dose effective concentration [28,30]. Comparison of intake of IMD between water intake and within food found that the administration in drinking water resulted in a 1.5 times higher intake of IMD compared with intake in a mixed diet. Thus, for these studies, a concentration of 7.5% (5% × 1.5 = 7.5%) was set as the high dose in order to obtain the same amount of IMD as 5% concentration did in drinking water. 3.75% was chosen as a half-dose of 7.5%. Control diet was made of standard chow (5L0D PicoLab Rodent diet, Labdiet, USA) with 7.5% *w*/*w* cellulose (CTRL). The low dose diet contained standard chow supplemented with 3.75% *w*/*w* IMD (LD IMD). The high dose diet contained standard chow supplemented with 7.5% *w*/*w* IMD (HD IMD). IMD was provided by Hayashibara Co., LTD. (Okayama, Japan). Each diet was prepared by mixing the powder form of 5L0D and the fiber blend with the addition of sterile water. The diet mix was then prepared in pellets and dehydrated at room temperature. Food consumption and mouse weights were monitored and measured weekly. Fecal samples were collected at baseline, six weeks, and twelve weeks and immediately snap frozen in liquid nitrogen. The samples were then stored at −80 °C for further analysis.

### 2.2. Histological Assessment

Segments of the terminal ileum and proximal colon were collected for histology. Both ileum and colon were flushed with phosphate-buffered saline containing 0.1% gentamycin and immediately fixed in 10% v/v neutral buffered formalin. The fixed samples were paraffin-embedded, sectioned, and stained with hematoxylin and eosin. Disease scoring methods used were as previously described [31] and based on four scores: (1) epithelial hyperplasia (0–3), (2) enterocyte injury (0–3), (3) lymphocytes (0–2) and neutrophil infiltration (0–2) in the lamina propria. The total maximum histological score (10) is based on the sum of the individual scores.

### 2.3. Colonic Cytokine Measurements

Snap frozen proximal colonic tissues were homogenized in phosphate-buffered saline (PBS) with 0.05% Tween 20. Cytokines (TNF-α, interferon gamma (IFN-γ), IL-1β, mouse Keratinocyte-derived Cytokine (mKC), IL-6, and IL-12 heterodimer p70 (IL-12p70)) were analyzed with the Multi-Spot electro-chemiluminescence system (Meso Scale Discovery, Rockville, MD, USA) per manufacturer’s protocol. IL-23 and IL-17 were measured with the Duoset ELISA (R&D biosystems, Minneapolis, MN, USA) according to the manufacturer’s instruction. Cytokine concentrations were normalized to tissue weight.

### 2.4. Plasma Lipopolysaccharide (LPS) Measurement

Blood samples were centrifuged at 1500× *g* for 10 min. Plasma was collected then diluted 20-fold in endotoxin free water, and LPS measured using Endotoxin (ET) Enzyme-Linked Immunosorbent Assay (ELISA) Kit (Abbexa Ltd., Cambridge, UK) as per the manufacturer’s protocol.

### 2.5. Fecal Lipocalin2 (Lcn2) Measurement

Fecal samples were processed as previously described [32] with PBS with 0.05% Tween 20. Lcn2 was quantified via the Lcn2/neutrophil gelatinase-associated lipocalin (NGAL) mouse ELISA kit (R&D biosystems, Minneapolis, USA) according to the manufacturer’s instructions. Lcn2 concentration was normalized to fresh stool weight.

### 2.6. Fecal Short Chain Fatty Acids (SCFAs) Measurement

The quantification of fecal SCFAs was performed using gas chromatography as previously described [33]. Briefly, 40 mg of fecal samples were homogenized in 300 μL 0.1 N hydrochloric acid followed by the addition of phosphoric acid (50 μL, 0.25%). Samples were centrifuged at 3000× *g* for 10 min. Internal standard solution (150 mg of 4-methyl-valeric acid, S381810, Sigma-Aldrich, Saint Louis, MO, USA) was added to the supernatant and SCFAs were analyzed via Varian model 3400 Gas Chromatograph (Varian, Walnut Creek, CA, USA) with a Stabilwax-DA column (30-m × 0.25-mm i.d.; Restek, Bellefonte, PA, USA).

### 2.7. DNA Extraction and 16S Ribosomal RNA (rRNA) Gene Sequencing Analysis

The DNA extraction method has been previously described [33]. Fecal microbial DNA was extracted with AquaStool solution (Multitarget Pharmaceuticals LLC, Colorado Springs, CO, USA) as per the manufacturer’s instruction. Briefly, 100 mg of mouse fecal pellet was homogenized in AquaStool solution with 0.1 mm beads at 0.6 m/s for 40 s. AquaRemove was added to remove potential Polymerase chain reaction (PCR) inhibitors per manufacturer’s instruction followed by ethanol/NaCl precipitation for further purification. DNA Samples were sent to Genome Quebec (McGill University, Montreal, Canada) for Illumina Miseq sequencing. V3-V4 region of universal 16S rRNA primers with 341 forward primer: 5′-TCG TCG GCA GCG TCA GAT GTG TAT AAG AGA CAG CCT ACG GGN GGC WGC AG-3′ and 805 reverse primer: 5′-GTC TCG TGG GCT CGG AGA TGT GTA TAA GAG ACA GGA CTA CHV GGG TAT CTA ATC C-3′ were used.

Demultiplexed paired-end sequences were merged and quality control implementation was performed (retention length without adapter = 235  bp, mean sequence quality score ≥ 30) and features table construction (amplicon sequences variants, ASVs) via DADA2 [34] plugin in QIIME2 (version 2019.10) [35]. Samples with greater than 14,604 sequencing reads were retained. To avoid analyzing spurious sequences, ASVs with an average relative abundance below 0.02% in all samples were removed, which had 471 ASVs remained. An even sequence depth of 12,572 reads per sample was used to conduct microbiome diversity and composition analyses. Taxonomy assignments from the phylum to genus levels were conducted by a pre-trained Naive Bayes classifier [36] (Silva 132 99% OTUs database) and the q2-feature-classifier function in QIIME2. Prior to statistical analysis, microbial counts at the phylum, genus, and ASV levels were transformed via centered log-ratio (CLR) for correcting sparse compositionality [37].

### 2.8. Bacterial Community Data Analysis

The analyses were performed using QIIME2 and R (version 3.5.1). Alpha-diversity of Shannon index and community balance of Pielou’s evenness index was conducted using the QIIME2. A repeated measures two-way Analysis of variance (ANOVA) model with Tukey post-hoc test was used to assess differences between times (Baseline, Week 6 and Week 12) or between treatment groups (CTRL, LD IMD and HD IMD), while between-group differences in shift (log2 transformed fold-change: Week12/Baseline) were determined by Analysis of covariance (ANCOVA) with permutations test (*n* = 999), adjusted by food consumption. Post hoc test was performed by Tukey′s procedure. To assess beta-diversity, Euclidean distance between samples was calculated from CLR transformed ASVs data using the R package vegan [38]. Principal coordinates analysis (PCoA) was conducted via R package ape [39]. Significant differences of beta-diversity between treatment groups were assessed via permutational multivariate analysis of variance (PERMANOVA, *n* = 999), adjusted by the food consumption using the Adonis function in vegan and pairwise comparison function in R package EcolUtils [40]. Comparisons of phyla and genera (CLR-transformed counts) between baseline and Week 12 in each treatment group were performed by paired Wilcoxon tests. Comparison between-group differences in CLR-transformed shift (Δ Week 12—Baseline) were determined by Kruskal-Wallis test, then unpaired Wilcoxon test was performed to identify pairwise comparisons. The *p*-values of Kruskal-Wallis test were adjusted by Benjamini-Hochberg false discovery rate (FDR) method and referred as *q*-values. Differences with *q*-values < 0.15 were considered significant.

### 2.9. Host Clinical and Immunometabolic Data Analysis

The analyses were performed using R and GraphPad Prism (version 8.0). For longitudinal analyses of the shifts of weight gain, food consumption and fecal SCFA concentrations, a repeated measures two-way ANOVA model with Tukey post-hoc test was used to assess differences between times or between groups. Area under the curve (AUC) was used to quantify food consumption. AUC was calculated from weeks 1 to 6 for each mouse, where higher values mean more food intakes. Comparison between-group differences in AUC of food consumption were determined by Kruskal-Wallis test with Dunn’s post-hoc test. Comparison between-group differences in shift (log2 transformed fold-change: Week12/Baseline) of SCFAs and weight gain were determined by ANCOVA with permutations test (*n* = 999), adjusted by food consumption. Comparison between-group differences in levels of immune marker at Week 12 were determined by ANCOVA with permutations test (*n* = 999), adjusted by food consumption. Permutation test was used to control the false positive error and family-wise error rate [41,42]. Differences with a permutation-based adjusted *p*-value < 0.05 were considered significant. The multivariate data of immune markers and contribution vectors were conducted by principal component analysis (PCA) in vegan. To assess the host-bacterial compositional interaction, Spearman’s correlation was performed. *p*-Values were adjusted using the Benjamini-Hochberg FDR, and considered as significant as *q*-values < 0.15 [43].

## 3. Results

### 3.1. Effects of IMD on Weight Gain and Food Consumption

**Figure 2:**
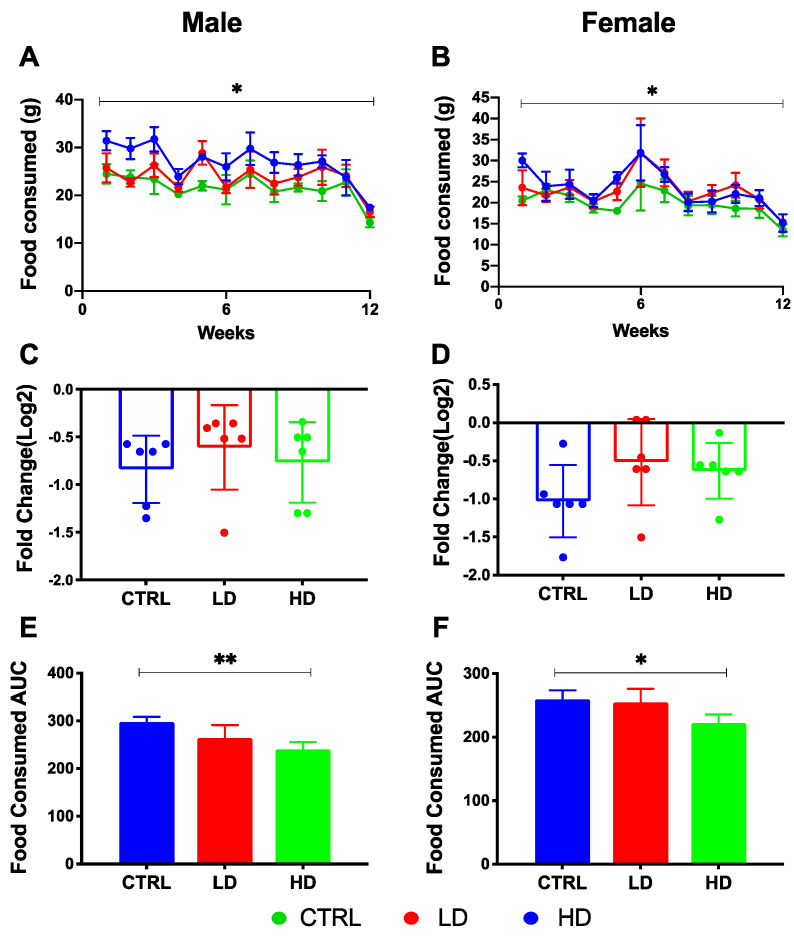
Food consumption of IL-10^−/−^ mice over 12 weeks of isomaltodextrin (IMD) intervention. Changes in weekly food consumption over 12 weeks are shown in (**A**) male mice and (**B**) female mice as Log2 fold change. Repeated measures two-way ANOVA with Tukey post-hoc test was used to assess differences between times or between treatments. Comparison of shifts in food intake (log2 transformed fold-change: Week12/Baseline) between treatment groups are shown in (**C**) male mice and (**D**) female mice. Comparison between-group differences in area under the curve (AUC) of food consumption are shown in (**E**) male mice and (**F**) female mice. Statistical between-group differences analysis used Kruskal-Wallis with Dunn′s test. ** *p* < 0.01, * *p* < 0.05. Data were reported as mean ± SD. Abbreviation: CTRL, Control diet; LD, Low dose IMD diet; HD, High dose IMD diet; AUC, area under the curve.

From baseline to Week 12, daily food consumption of both male and female mice decreased in all diet groups (*p* < 0.05; Figure 2A,B). Although the comparison of shifts in food intake (log2 transformed fold-change: Week12/Baseline) between treatment groups had no difference between groups (Figure 2C,D), the high-dose IMD group showed a significantly lower AUC value for 12 weeks of food consumption than did the control group in both male and female mice (*p* = 0.002 and *p* = 0.005; Figure 2E,F) indicating a lower food consumption in mice receiving the highest dose of IMD. Therefore, to correct the effect of IMD on host health outcomes and microbiota, the food consumption AUC was included as a covariate in further comparison analyses among groups. Body weight of both male and female mice increased over the 12-week experimental period in all diet groups (*p* < 0.01; Figure S1). There was no difference in weight gain between mice receiving IMD supplementation and mice in the control group in either male or female mice.

### 3.2. Effects of IMD on Levels of Inflammation-Related Markers at Week 12

**Figure 3:**
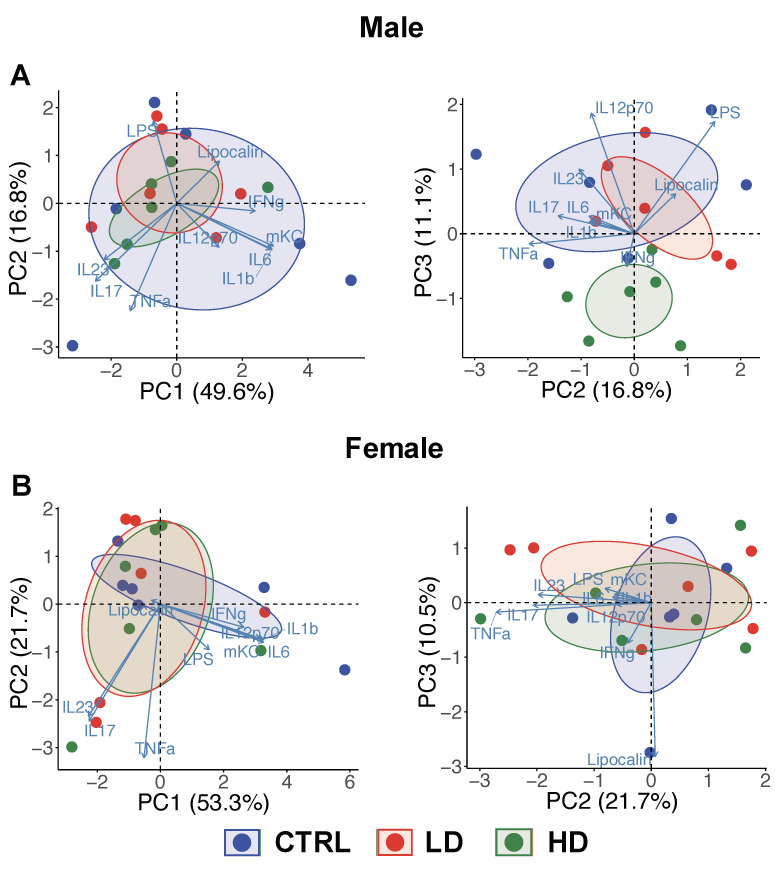
The isomaltodextrin (IMD) intervention association with the variability of inflammatory responses in male IL-10^−/−^ mice at Week 12. Principal component analysis (PCA) plot shows a 2-dimensional scatter plot (PC1 vs. PC2; PC2 vs. PC3) comparing the variation of inflammation between control (CTRL), low-dose IMD (LD) and high-dose IMD (HD) in (**A**) male and (**B**) female mice, respectively.

In male mice, upon completion of the 12-week intervention, PCA plots done using the inflammatory variables identified in Table 1 demonstrated a separation between the high-dose IMD group and the control group on PC3 axis with 11.1% variation explanation among the subjects (Figure 3A, PC2 vs. PC3). PCA analysis also identified IL-12p70, LPS and TNF-α as the top three variables contributing to this variation. In contrast, female mice did not separate across the various treatment groups in the PCA plots based on inflammatory markers (Figure 3B). Of the individual marker comparisons, colonic IL-12p70 was significantly depressed in the male mice given high-dose IMD compared to the control group (*p* = 0.04; Table 1). These results indicate both a sex- and dose-dependence of effects of IMD supplementation in mice.

Statistical comparisons between-groups were tested by ANCOVA and considered the food consumption AUC as a covariate. Permutation test (*n* = 999) was used to control the false positive error and family-wise error rate. The permutation-adjusted *p*-values were reported. Tukey’s post hoc test was performed to assess pairwise comparisons of each inflammatory marker. a indicates a significant difference with the control (*p* < 0.05). Data were reported as mean ± SD. Abbreviation: IL, interleukin; IFNγ, Interferon-gamma; TNFα, Tumor Necrosis Factor alpha; mKC, mouse Keratinocyte-derived Cytokine; LPS, Lipopolysaccharide.

### 3.3. Histological Scoring

After 12 weeks, the IMD and control groups showed no significant differences in histology scores, including neutrophil infiltration, lymphocyte infiltration and tissue injury, among both male and female IL-10^−/−^ mice (Table S1). 

### 3.4. Effect of IMD on Fecal Microbiota Composition and Fecal Short Chain Fatty Acids

#### 3.4.1. Beta-Diversity

**Figure 4:**
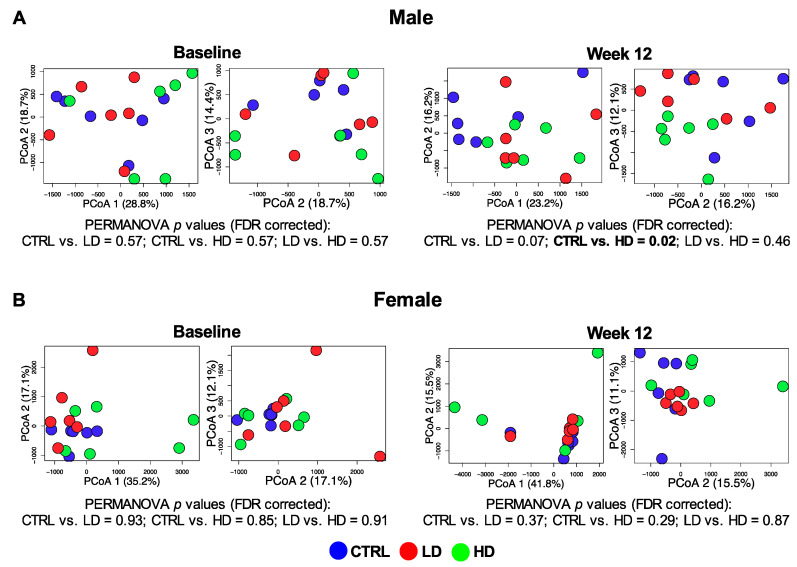
Comparison of fecal microbiota beta-diversity. Principal coordinates analysis (PCoA) plots showing: (**A**) male mice and (**B**) female mice microbiota dissimilarity (inter-subject beta-diversity) between control (CTRL), low-dose IMD (LD) and high-dose IMD (HD) at baseline and at week 12, respectively. The significant differences were assessed by Adonis PERMANOVA using Euclidean distance and adjusted by food consumption AUC. The multiple comparisons were corrected by FDR.

Baseline beta-diversity of fecal microbiota was similar between groups (*q* = 0.57; Figure 4). After the 12-week intervention, the high-dose IMD and control groups demonstrated significantly different beta-diversity in male mice (*q* = 0.03; Figure 4A) but not in female mice (*q* = 0.29; Figure 4B).

#### 3.4.2. Alpha-Diversity

**Figure 5:**
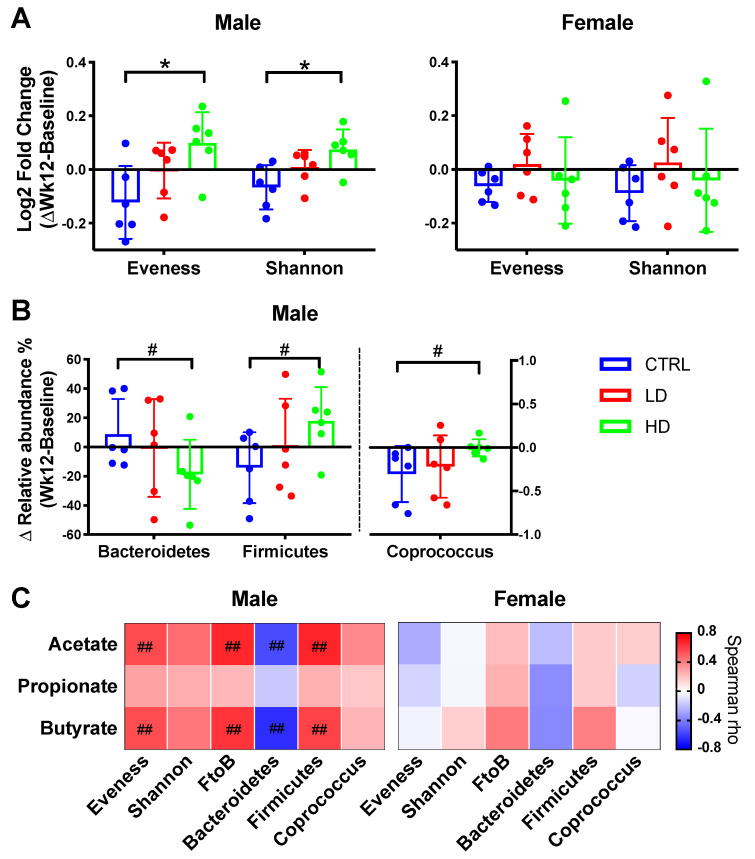
Summary of significant changes in fecal microbiota composition over 12 weeks of isomaltodextrin (IMD) supplementation. (**A**) Comparison of shifts in fecal alpha-diversity (log2 fold-change) between control (CTRL), low-dose IMD (LD) and high-dose IMD (HD). Statistical comparisons between-groups were by ANCOVA with permutations (*n* = 999), adjusted by the food consumption area under the curve (AUC). Tukey′s post hoc test was performed. * *p* < 0.05. (**B**) Comparison of shifts in bacterial relative abundance. Comparison results of all bacterial taxa at phylum and genus level are shown in Table S2. This figure presents a summary of the significant statistics in Table S2. Statistical comparisons between-groups were by Kruskal–Wallis test with FDR correction (Table S2). ^#^ *q* < 0.15. Data were reported as mean ± SD. (**C**). Associations between shift of microbial composition and shift of short chain fatty acid concentrations (ΔWk12-BL) were assessed by Spearman rank correlation with FDR correction. ^##^ *q* < 0.05, ^#^ *q* < 0.15.

In both male and female mice, the microbial Shannon diversity was significantly reduced in the control group (*p* = 0.03 and *p* = 0.009, respectively; Figure S2). Interestingly, only the male mice given high-dose IMD demonstrated a significant increase in Shannon diversity (*p* = 0.046). Moreover, in male mice, microbial evenness markedly increased in the high-dose IMD group over 12 weeks (*p* = 0.007) but decreased in the control group (*p* = 0.018; Figure S2A). The high-dose-IMD-induced changes in both evenness and Shannon diversity over 12 weeks also significantly differed from the control group (*p* = 0.01 and *p* = 0.01, respectively; Figure 5A). Together, this suggests that IMD can mitigate changes in microbial richness and balance caused by colitis development. Of note, these responses were only displayed in the male mice. In the female mice, IMD treatment did not increase evenness or Shannon diversity (*p* ≥ 0.3; Figure S2B). There was also no difference in changes between the IMD and control groups in female mice (*p* ≥ 0.39; Figure 5A).

#### 3.4.3. Bacterial Taxa Relative Abundance

IMD intervention also resulted in bacterial taxa relative abundance changes in male mice. At the phylum level, the changes among Firmicutes, Bacteroidetes and Firmicutes/Bacteroidetes ratios in the high-dose IMD treatment group were the reverse of those changes in the control group (*q* ≤ 0.11; Figure 5B and Table S2). At the genus level, the control group with colitis development demonstrated reduced relative abundance of *Coprococcus*, while the high-dose IMD treatment seemed to maintain this abundance level (*q* = 0.08). Similar to the diversity findings, these differences in bacterial taxa abundance were only found in the male mice with high-dose IMD. In the female group, there was no significant difference in shifts of bacteria taxa between the IMD and control groups (*q* ≥ 0.38; Table S3). Together, the results reveal that the impacts of IMD on microbial composition, including diversity and certain taxa abundance, were sex-specific and dose-dependent.

#### 3.4.4. Short Chain Fatty Acids

There were no significant differences in levels of individual fecal SCFA in any of the groups over the 12 weeks (Figures S3 and S4). However, further assessment of associations between SCFA levels and the microbiota showed that shifts in community evenness and Firmicutes/Bacteroidetes ratio showed strong positive correlations with shifts of both acetate and butyrate concentration, respectively (r_s_ ≥ 0.55, *q* < 0.05; Figure 5C). Of note, the significant correlations were only detected in male mice.

#### 3.4.5. Colonic Mucosal IL-12p70 Level Was Associated with IMD-Induced Microbiota Compositional Changes

**Figure 6:**
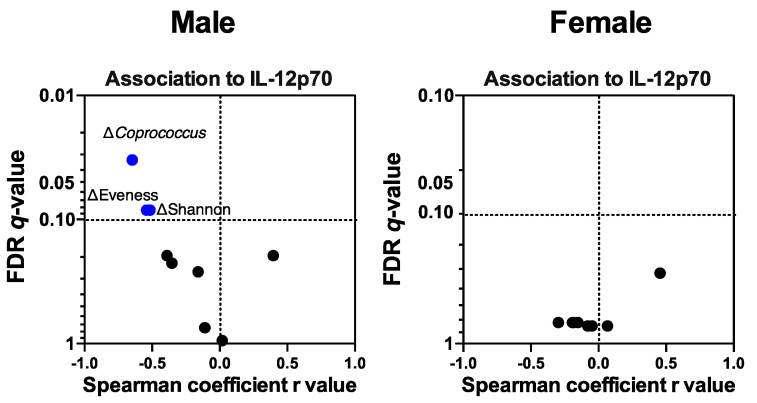
Association between bacterial compositional shifts with colonic mucosal IL-12p70 levels in male and female IL-10^−/−^ mice. Scatter plots show the association between shifts of bacterial taxa, alpha-diversity and short chain fatty acids (ΔW12–BL) over the 12 weeks IMD intervention and the IL-12p70 response at Week 12. Statistically significant and coefficient were assessed by Spearman rank correlation with false discovery rate (FDR) correction. Only significant correlations (*q* < 0.15) were shown the variable names. IL, interleukin.

Because the IL-12p70 level in male mice at the end of the intervention was lower in the IMD group than in the control group, we further assessed whether microbial compositional and functional shifts were linked to the IL-12p70 level. In the male group, the IL-12p70 level was negatively associated with increased evenness, Shannon diversity and the relative abundance of *Coprococcus* (r_s_ ≤ −0.51, *q* ≤ 0.08; Figure 6). Thus, under IMD supplementation, increased community diversity and *Coprococcus* proportion may contribute to reduced IL-12p70 levels. These significant associations were not detected in the female group, which suggested a sex difference in the interactions of IL-12p70 and microbiota.

## 4. Discussion

In this study we demonstrate that IMD supplementation fed to IL-10^−/−^ mice altered microbial diversity and taxa composition and immune profiles in both a dose- and sex-dependent fashion with male, but not female, mice responding. There is intense interest in using dietary manipulation to stimulate therapeutic microbiome changes and help to prevent or alleviate IBD [44,45,46]. Previous studies have shown that IBD patients exhibit reduced microbial diversity, recognized as a dysbiosis characterization [9,47,48,49]. Microbial communities with higher diversity and balance are considered more resilient and better able to efficiently exploit resources, including dietary fiber [50,51]. Consequently, higher diversity microbiomes provide less opportunity for pathogenic organism invasions and produce multiple metabolites that profoundly influence intestinal epithelial and immune function [52,53,54]. High fiber foods and fiber supplements have shown some promise in reducing inflammation in CD patients, but results have been inconsistent [55,56,57,58]. Mucosal immune function and susceptibility to IBD are also distinct between males and females [23,59], which suggests that sex differences are a prominent factor to be considered when tailoring therapy [60]. 

In male mice, IMD increased alpha-diversity, including community richness and evenness, and these changes positively correlated with fecal butyrate and acetate concentrations. At the genus level, the dominant effect of IMD was directed towards *Coprococcus.* Fecal and mucosal *Coprococcus* measurements are consistently reported as being decreased in IBD patients compared to healthy controls [61,62,63]. Depletion of this genus is therefore considered a signature of unbalanced microbial composition in IBD. Our results indicated that IMD supplementation, compared to the control, could help mitigate *Coprococcus* depletion. IMD is a branched polymer formed by a backbone of α-D-glucose, majorly linked by α-1,4 and α-1,6 glycosidic linkages [27]. Species of *Coprococcus*, such as *Coprococcus catus*, have been identified as having genes that encode α-glucose-degrading glycosidase (e.g., α-glucan phosphorylases [64] and α-1,4 glycosyltransferase [65]). Thus, it could be explained that IMD supplementation provides resources useful for *Coprococcus* which possesses the traits to access the highly α-glucan branched structure and competitively utilizes enough nutrient niches to maintain its abundance in the ecosystem. Our results differed from those obtained by Nishimura et al. with regards to changes at the phyla level [66]. However, changes induced by dietary interventions are highly influenced by the baseline microbiota; thus, it is not surprising that animals raised under different conditions show diverse changes in microbial composition in response to dietary fiber. 

We further identified that, after the 12-week intervention, IL-12p70 was lower in the high IMD group than in the control group. IL-12, including IL-12p70 and its subunit IL-12p40, are cytokines that can induce T helper-1, T helper-17, or innate lymphoid cell expansion in human IBD, leading to inflammation [67,68,69]. The association analysis revealed that changes in the microbiota, including the promotion of community diversity and the relative abundance of *Coprococcus*, were negatively associated with IL-12 levels. This suggested that IMD-induced alterations in microbial diversity and taxa compositions played a beneficial role in controlling IL-12 and might contribute to anti-inflammatory responses. Although previous studies in the DSS-induced colitis mouse model demonstrated anti-inflammatory effects of IMD with reduction of TNF-α and IL-6 [28,70], we did not see any effect on these cytokines. One possible reason is that different animal host genetic, environmental factors, and methods for inducing colitis would lead to unique interactions in the gastrointestinal system; a problem which continues to influence therapy reproducibility [71,72]. In addition, in contrast to the previous models which used water as a control [28,70], we applied cellulose, a non-fermentable fiber, as a placebo control to investigate the effect of fermentable IMD on colitis development. Recently, cellulose supplementation has been reported to prevent gut inflammation in the DSS-induced colitis mice model [73]. Thus, we speculate that the reason that IMD did not demonstrate greater anti-inflammatory effects on immune markers except for IL-12, compared to the control mice receiving cellulose, might be due to cellulose also having anti-inflammatory properties.

Interestingly, the significant IMD effects on microbiota diversity and composition and IL-12 were only found in the males. In contrast, the female mice receiving IMD displayed a reduction in alpha-diversity and *Coprococcus* abundance, as well as a high level of IL-12, as did the control group. The mechanisms underlying the sex modulation of effects of dietary fiber are uncertain. One possible reason, however, might be linked to sex hormones. A published landmark animal study discovered that sex hormones are associated with host immunity and can modulate microbiota community diversity and the relative abundance of bacterial taxa including *Coprococcus* [23]. Another more recent study found that *Coprococcus* was reduced by a high-fat diet in male mice, but no change was observed in female mice, a finding which suggested a possible association with sex hormones [22]. These studies importantly indicated that certain gut microbiota composition features exist in a sex dependent manner. The findings of our current research did not directly identify the mechanisms driving either fiber or sex effects. Instead, we have determined, for the first time, that the effects of dietary fiber on potentially hormone-dependent microbiota features, such as *Coprococcus*, were indeed sex-specific. Moreover, we found that the association with dietary fiber and the response of the inflammation marker IL-12 response were also sex-dependent indicating that differences in sex likely influence diet–microbe–immune interactions. This highlights the urgent need to identify and explain these mechanisms and how they interact.

The dose-response study design allowed us to identify microbial diversity and composition and colonic immune functions that exhibited dose-dependent responses to IMD. In the males, the dominant effects of IMD were only detected in the higher feeding dose group. We speculate that the lower amount of IMD supplemented was insufficient for providing essential nutrients in the gut ecosystem for taxa to reach or maintain high growth rates. Considering the strong associations between the shifts of microbial features and IL-12 response, it is possible to explain why low-dose IMD had no effect on the IL-12 response compared to the high-dose IMD. Further work is required to identify the dose necessary for IMD to maximize the therapeutic effect on the microbiome and inflammation while optimizing IBD patient tolerance and compliance. 

## 5. Conclusions

In conclusion, high dose IMD supplementation promoted global changes in microbiota diversity and taxa composition in male mice and mitigated colitis-induced gut dysbiosis and some immune changes. However, the effects were dose and sex dependent, with the positive changes seen only in male mice with high dose supplementation. These findings provide one possible factor that may help explain why efficacy of dietary fiber supplements in IBD is inconsistent among interventional studies. Future studies are needed to identify dose-responses and tolerance in human trials for reaching maximum therapeutic effect. Further, if fiber and sex interactions are seen in patients with IBD, therapeutic supplementation with specific dietary fibers might not work equally well for IBD patients of both sexes. Future IBD studies should account for sex effects and interactions with other environmental factors in trials and assess for potential sex-mediated differences in clinical outcomes.

## Figures and Tables

**Table 1:** Inflammation-related marker levels of IL-10^−/−^ mice receiving isomaltodextrin (IMD) intervention at Week 12.

	Male			*p*	Female			*p*
	Control	LD	HD		Control	LD	HD	
**Markers of colonic inflammation**								
IL1β (ng/g)	11.0 ± 15.7	4.65 ± 6.78	3.52 ± 7.30	0.43	13.9 ± 18.7	4.95 ± 11.4	6.87 ± 12.1	0.56
IL6 (ng/g)	0.40 ± 0.55	0.21 ± 0.30	0.14 ± 0.31	0.62	1.01 ± 1.55	0.30 ± 0.73	0.40 ± 0.84	0.48
IL12p70 (ng/g)	0.15 ± 0.12	0.09 ± 0.11	<0.01 ± 0.0 ^a^	0.04	0.23 ± 0.39	0.04 ± 0.11	0.11 ± 0.18	0.51
IL17 (ng/g)	24.0 ± 15.6	23.3 ± 11.9	25.3 ± 9.17	1.00	21.4 ± 10.0	26.3 ± 18.9	25.7 ± 20.5	0.88
IL23 (ng/g)	12.9 ± 6.01	12.8 ± 7.57	11.5 ± 2.89	0.92	10.7 ± 5.29	14.7 ± 10.2	15.3 ± 13.8	0.94
IFNγ (ng/g)	0.02 ± 0.02	0.01 ± 0.01	0.03 ± 0.03	0.42	0.04 ± 0.05	0.03 ± 0.07	0.02 ± 0.02	0.92
TNFα (ng/g)	1.09 ± 0.72	1.02 ± 0.27	1.26 ± 0.39	0.69	0.91 ± 0.39	1.13 ± 0.64	1.04 ± 0.54	0.68
mKC (ng/g)	1.50 ± 1.70	0.82 ± 0.85	0.69 ± 0.77	0.41	2.85 ± 3.76	0.91 ± 1.54	1.47 ± 2.02	0.41
Lipocalin2 (ng/g)	180 ± 68.5	192 ± 51.2	151 ± 70.8	0.60	188 ± 161	125 ± 74.6	185 ± 84.1	0.57
**Markers of systemic inflammation**								
LPS (EU/mL)	9.41 ± 2.05	9.72 ± 0.58	8.49 ± 1.43	0.43	8.09 ± 0.67	8.58 ± 1.80	8.83 ± 1.58	0.71

Statistical comparisons between-groups were tested by ANCOVA and considered the food consumption AUC as a covariate. Permutation test (*n* = 999) was used to control the false positive error and family-wise error rate. The permutation-adjusted *p*-values were reported. Tukey’s post hoc test was performed to assess pairwise comparisons of each inflammatory marker. ^a^ indicates a significant difference with the control (*p* < 0.05). Data were reported as mean ± SD. Abbreviation: LD, Low dose IMD diet; HD, High dose IMD diet; IL, interleukin; IFNγ, Interferon-gamma; TNFα, Tumor Necrosis Factor alpha; mKC, mouse Keratinocyte-derived Cytokine; LPS, Lipopolysaccharide.

## Supplementary Materials

The following are available online at https://www.mdpi.com/2072-6643/12/7/2088/s1, Figure S1: Weight gain of IL-10^−/−^ mice over 12 weeks isomaltodextrin (IMD) intervention, Figure S2: Changes in fecal microbiota alpha-diversity of IL-10^−/−^ mice over 12 weeks isomaltodextrin (IMD) intervention, Figure S3: Changes in fecal short chain fatty acids (SCFAs) concentration of IL-10^−/−^ male mice over 12 weeks isomaltodextrin (IMD) intervention, Figure S4: Changes in fecal short chain fatty acids (SCFAs) concentration of IL-10^−/−^ female mice over 12 weeks isomaltodextrin (IMD) intervention, Table S1: Histology scoring assessment in male and female IL-10^−/−^ mice with isomaltodextrin (IMD) for 12 weeks, Table S2: Fecal bacterial relative abundance changes in male IL-10^−/−^ mice with isomaltodextrin (IMD) intervention, Table S3: Fecal bacterial relative abundance changes in female IL-10^−/−^ mice with isomaltodextrin (IMD) intervention.
