# Supplementary material for: Sex-Specific Differences in the Gut Microbiome in Response to Dietary Fiber Supplementation in IL-10-Deficient Mice
Source: Nutrients. 2020 Jul 15;12(7):2088. doi: 10.3390/nu12072088 (PMC7400915; doi:10.3390/nu12072088)
Supplement: Supplementary file 1 [file nutrients-12-02088-s001.pdf]

SUPPLEMENTAL MATERIALS

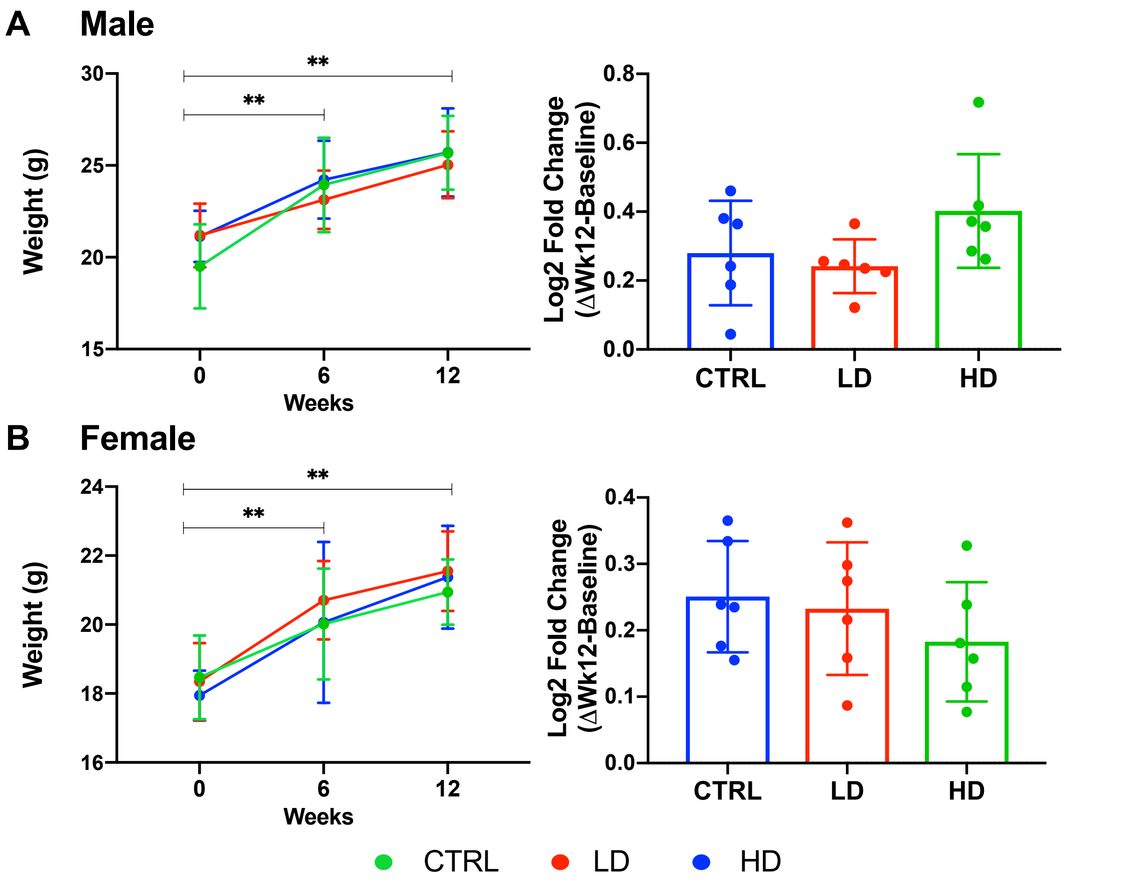

Figure S1. Weight gain of IL-10-/- mice over 12 weeks isomaltodextrin (IMD) intervention. The changes in weight over 12 weeks, and comparison of shifts in weight (log2 transformed fold-change: Week12/Baseline) between treatment groups in (**A**) male mice, and (**B**) female mice, respectively. Repeated measures two-way ANOVA with Tukey post-hoc test was used to assess differences between times or between treatments. Comparison of shifts in weight (Δ Week 12 – Baseline) between treatments groups was used Kruskal-Wallis with Dunn's test. ** $p < 0.01$, * $p < 0.05$. Data were reported as mean ± SD.

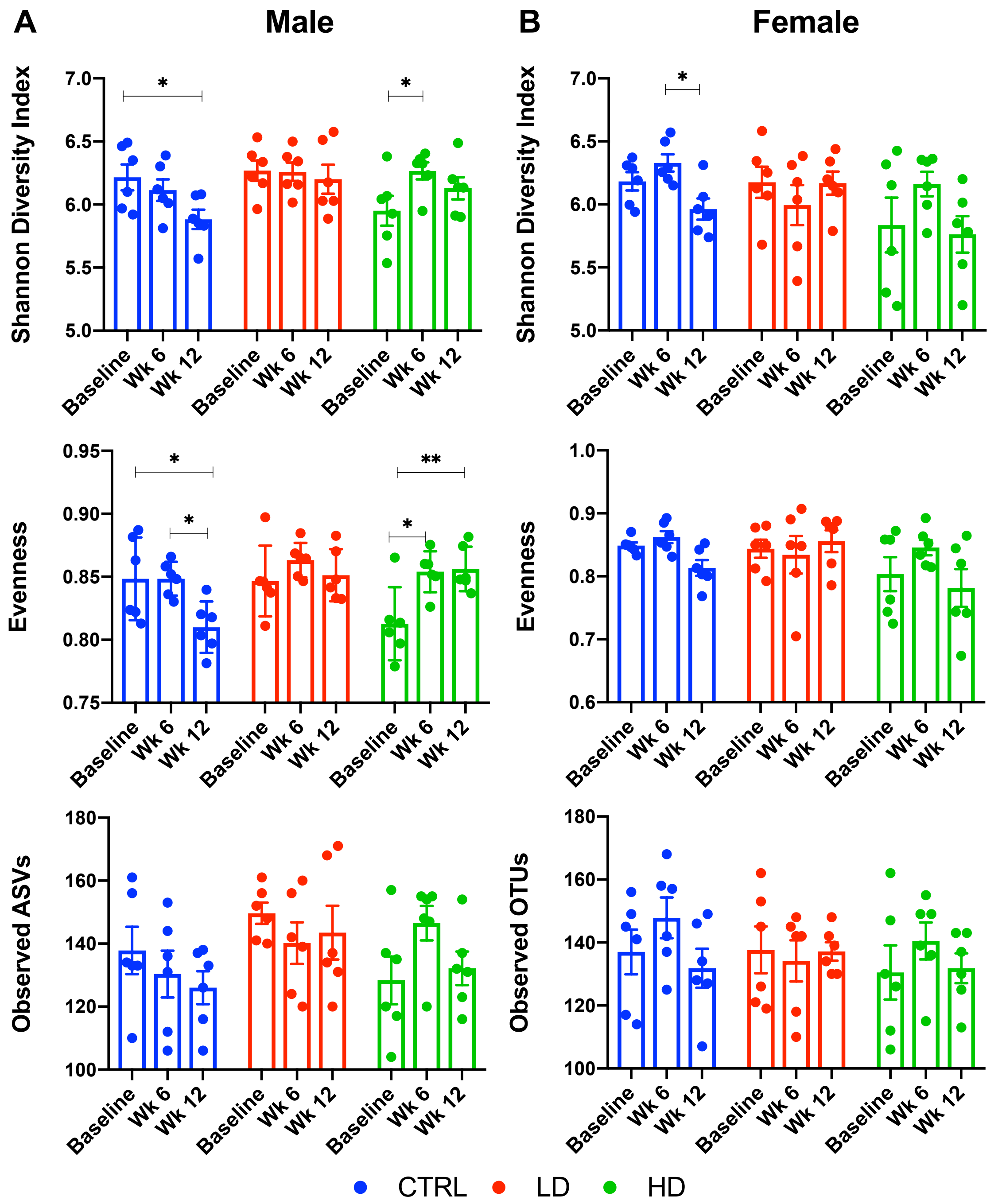

Figure S2. Changes in fecal microbiota alpha-diversity of IL-10-/- mice over 12 weeks isomaltodextrin (IMD) intervention. The changes in alpha-diversity index (Shannon, Evenness and Observed ASVs) over 12 weeks among the treatment groups in (**A**) male mice, and (**B**) female mice, respectively. Repeated measures two-way ANOVA with Tukey post-hoc test was used to assess differences between times or between treatments. ** $p < 0.01$, * $p < 0.05$. Data were reported as mean ± SD.

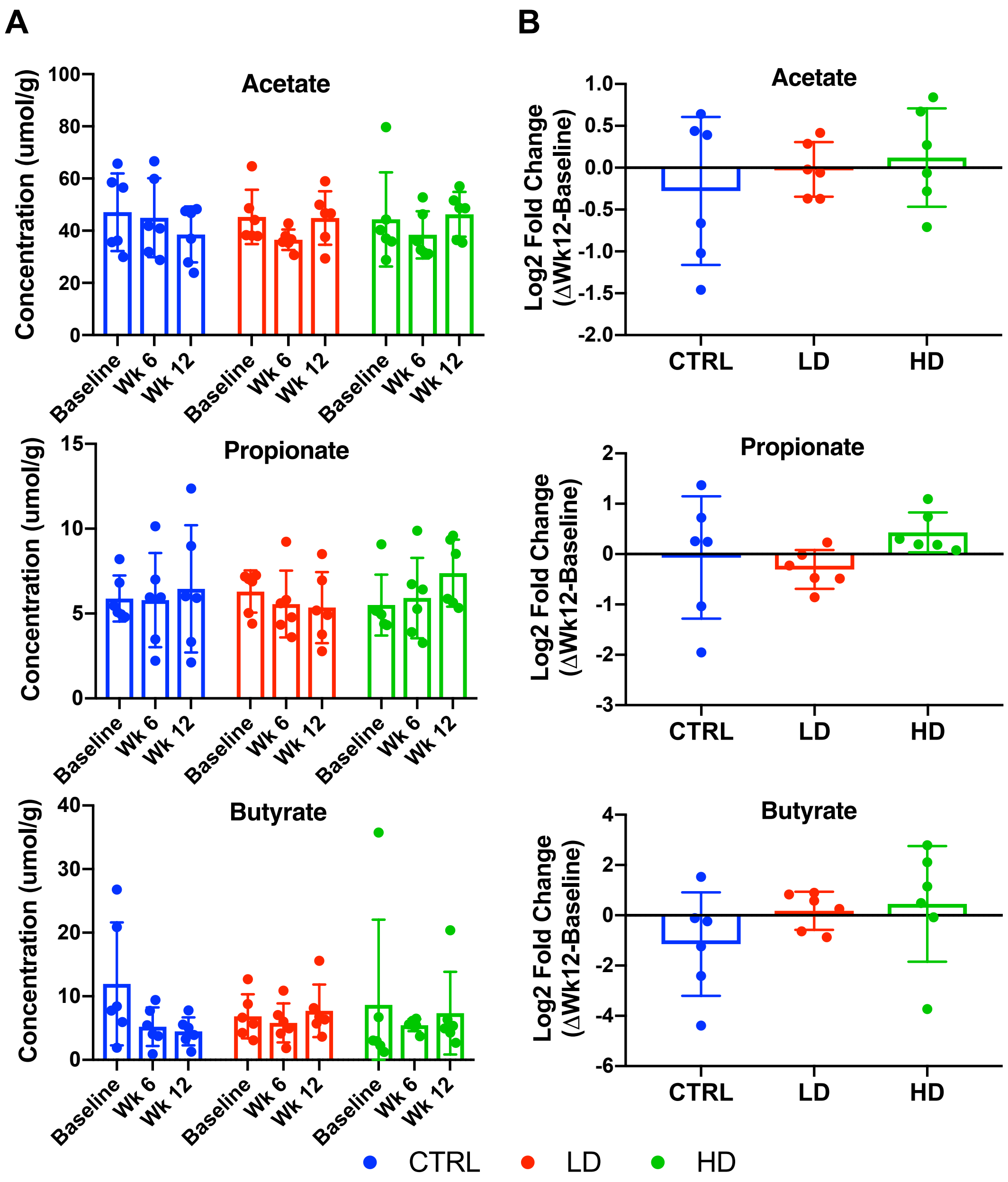

Figure S3. Changes in fecal short chain fatty acids (SCFAs) concentration of IL-10-/- male mice over 12 weeks isomaltodextrin (IMD) intervention. (**A**) Repeated measures two-way ANOVA with Tukey post-hoc test was used to assess differences between times or between treatments. (**B**) Comparison of shifts in SCFAs concentrations (Δ Week 12 – Baseline) between treatments groups was conducted by ANCOVA with permutations (n=999), adjusted by the food consumption AUC. Post hoc test was performed by Tukey's procedure. Data were reported as mean ± SD.

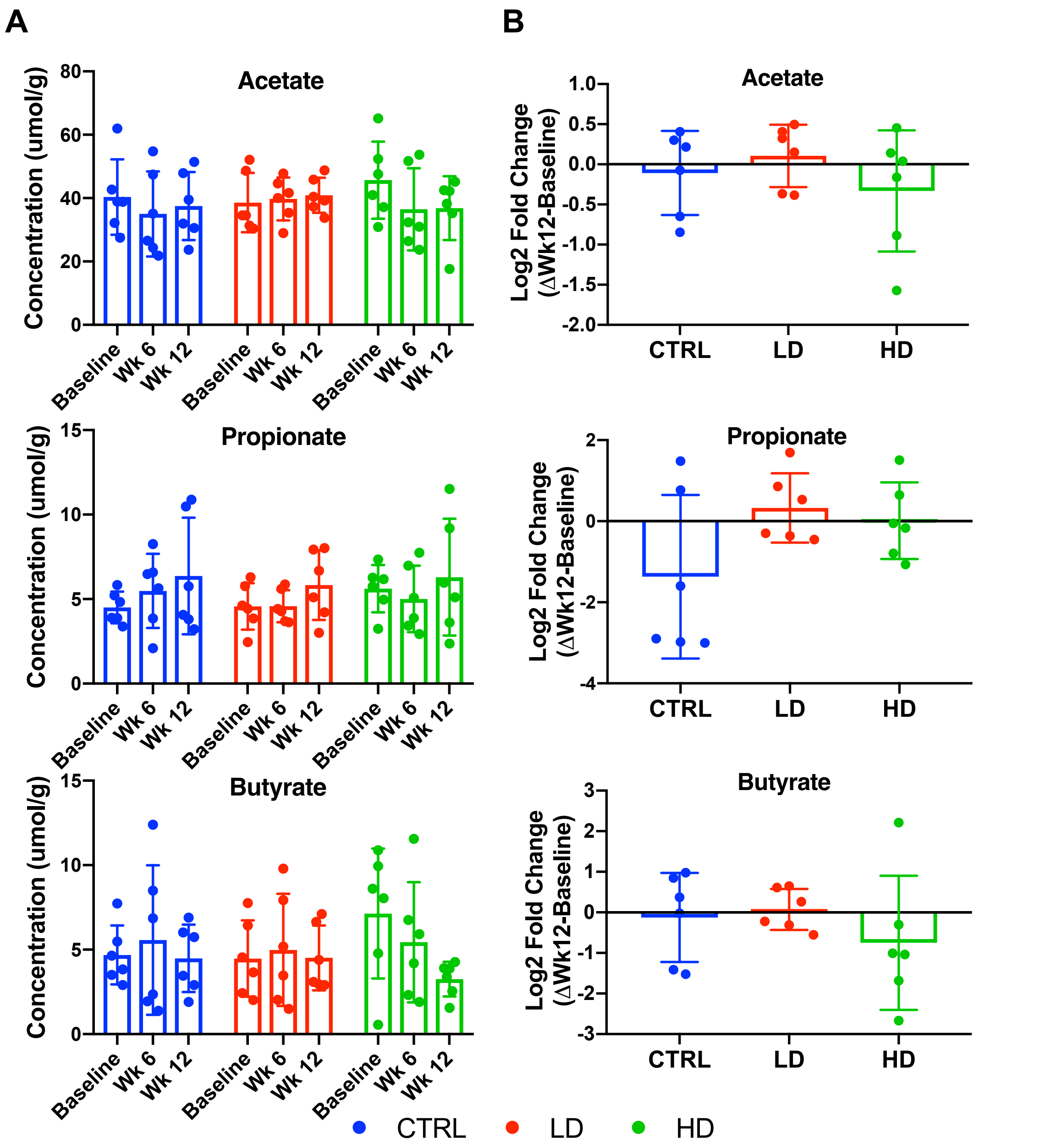

Figure S4. Changes in fecal short chain fatty acids (SCFAs) concentration of IL-10-/- female mice over 12 weeks isomaltodextrin (IMD) intervention. (**A**) Repeated measures two-way ANOVA with Tukey post-hoc test was used to assess differences between times or between treatments. (**B**) Comparison of shifts in SCFAs concentrations (Δ Week 12 – Baseline) between treatments groups was used ANCOVA with permutations (n=999), adjusted by the food consumption AUC, and with Tukey post-hoc test. ** $p < 0.01$, * $p < 0.05$. Data were reported as mean ± SD.

Table S2. Fecal bacterial relative abundance changes in male IL-10$^{-/-}$ mice with isomaltodextrin (IMD) intervention

Taxa	Control					Low Dose IMD					High Dose IMD					Between Group Shift *p* values	FDR
	Baseline	Week 12	Absolute Shift	Within Group *p* values	FDR	Baseline	Week 12	Absolute Shift	Within Group *p* values	FDR	Baseline	Week 12	Absolute Shift	Within Group *p* values	FDR		
**Phylum**																	
F/B Ratio	0.94±0.7	0.52±0.4	-0.42±0.7	0.44	0.56	0.91±0.5	1.27±1.4	0.36±1.8	1.00	1.00	0.42±0.3	1.03±0.8	0.61±1	0.22	0.58	0.03	**0.10***
Bacteroidetes	55.52±19.6	64.3±16.7	8.79±24.1	1.00	1.00	52.71±13.7	52.02±20.6	-0.68±33	0.84	1.00	71.05±12.8	52.34±14.6	-18.7±23.7	0.22	0.58	0.03	**0.10***
Firmicutes	41.79±18.4	27.67±13.7	-14.1±24.3	0.44	0.56	42.32±12.9	42.84±23.6	0.52±32.6	1.00	1.00	26.62±12.8	44.51±14.5	17.88±23.1	0.16	0.58	0.04	**0.11***
Verrucomicrobia	0±0	3.89±4.1	3.89±4.1	0.06	0.28	2.12±5.2	2.51±6.1	0.38±0.9	0.37	0.74	0.64±1.3	1.23±2.7	0.6±3.3	0.79	1.00	0.15	0.29
Proteobacteria	0.96±0.2	3.3±2.9	2.34±2.8	0.06	0.28	0.81±0.3	1.57±0.8	0.76±0.7	0.03	0.25	0.84±0.5	1.3±1	0.46±1.4	0.44	0.88	0.18	0.29
Tenericutes	1.47±2.3	0.64±0.6	-0.82±1.7	0.36	0.56	1.69±2.8	0.56±0.7	-1.12±2.8	0.31	0.74	0.33±0.7	0.18±0.3	-0.15±0.9	1.00	1.00	0.57	0.76
Cyanobacteria	0.03±0.1	0±0	-0.03±0.1	1.00	1.00	0±0	0±0	0±0	NA	NA	0±0	0±0	0±0	NA	NA	NA	NA
Actinobacteria	0.07±0.1	0.16±0.1	0.09±0.1	0.10	0.30	0.23±0.1	0.26±0.2	0.03±0.2	0.28	0.74	0.27±0.2	0.25±0.2	-0.02±0.4	0.84	1.00	0.69	0.79
TM7	0.18±0.2	0.05±0.1	-0.13±0.2	0.28	0.56	0.12±0.1	0.23±0.2	0.11±0.2	1.00	1.00	0.24±0.2	0.18±0.2	-0.06±0.3	0.22	1.00	0.94	0.94
**Genus**																	
f_S24-7	39±14.6	41.24±11.2	2.25±19.6	0.84	0.89	37.29±13.4	34.43±17.6	-2.86±28	1.00	1.00	42.28±8.5	27.93±14.1	-14.3±15.5	0.09	0.61	0.24	0.68
f_Lachnospiracea_1	21.1±6.8	11.99±7	-9.1±12.2	0.22	0.40	21.54±5.4	14.79±10.1	-6.75±12	0.56	0.79	13.55±4.9	20.85±5.9	7.3±9	1.00	1.00	0.09	0.54
g_[Prevotella]	4.8±3.9	6.52±5.5	1.72±6.5	1.00	1.00	3.51±2	3.39±2.4	-0.12±2.3	0.56	0.79	9.26±5	7.43±3.2	-1.83±7.1	0.31	0.78	0.94	0.96
o_Clostridiales_1	5.91±3.3	2.78±1.3	-3.14±4.1	0.22	0.40	6±2.5	7.86±4.8	1.86±4.9	0.56	0.79	3.83±3.2	8.5±3	4.67±5	0.22	0.70	0.09	0.54
g_Prevotella	2.77±2.7	9.36±4.6	6.6±5.3	0.03	0.27	2.45±1.2	6.28±4.8	3.83±5.1	0.56	0.79	7.67±7	6.68±4.3	-0.99±6.8	0.84	0.95	0.09	0.54
f_Rikenellaceae	5.42±2.5	2.6±1.2	-2.82±3.1	0.16	0.40	5.89±4.8	4.17±4.7	-1.73±7.1	0.56	0.79	7.73±4.6	3.43±2.8	-4.3±4.4	0.09	0.61	0.59	0.76
g_Bacteroides	1.32±0.4	2.67±1.2	1.35±1	0.31	0.46	1.22±1.1	2.82±2.4	1.6±2.9	0.16	0.68	2.58±2	5.89±5	3.31±4.1	0.84	0.95	0.31	0.68
g_Oscillospira	3.05±1.9	2.29±0.9	-0.76±2.3	0.44	0.57	4.42±3.2	2.67±2	-1.76±4.5	0.56	0.79	1.81±0.9	2.63±1.6	0.82±2.2	0.56	0.95	0.59	0.76
g_Akkermansia	0±0	3.89±4.1	3.89±4.1	0.03	0.27	2.12±5.2	2.51±6.1	0.38±0.9	0.31	0.79	0.64±1.3	1.23±2.7	0.6±3.3	1.00	1.00	0.13	0.66
f_Lachnospiraceae_2	3.84±2.7	1.6±1.4	-2.24±3	0.06	0.31	2.1±1.5	5.52±6.7	3.42±7.5	0.31	0.79	2.38±2.1	1.93±1.3	-0.45±3	0.31	0.78	0.48	0.76
g_Lactobacillus	0.97±2	4.17±8.8	3.19±6.8	0.31	0.46	1.6±2.3	6.67±6.8	5.07±6.7	0.09	0.47	1.38±1	3.2±2.9	1.81±2.8	0.69	0.95	0.24	0.68
o_Clostridiales_2	3.11±2.5	0.83±0.5	-2.28±2.6	0.31	0.46	3.64±2.5	1.21±1.2	-2.43±2.3	0.06	0.36	1.36±1.2	1.81±2.1	0.45±2.7	1.00	1.00	0.70	0.79
g_AF12	2.14±1.1	1.09±0.8	-1.05±1.1	0.16	0.40	2.28±1.8	0.89±0.9	-1.39±1.7	0.06	0.36	1.4±2.3	0.45±0.8	-0.95±2.5	0.16	0.61	0.70	0.79
g_[Ruminococcus]	1.2±0.9	2.73±3.7	1.53±3.7	0.84	0.89	0.97±0.5	1.99±3	1.02±3.2	0.69	0.89	0.72±0.4	3.53±4.6	2.82±4.6	0.69	0.95	0.70	0.79
g_Sutterella	0.22±0.2	1.39±1	1.17±1.1	0.16	0.40	0.39±0.5	0.93±1.1	0.54±1.2	0.84	1.00	0.23±0.3	0.5±0.6	0.27±0.8	0.44	0.85	0.70	0.79
g_Anaeroplasma	1.47±2.3	0.64±0.6	-0.82±1.7	0.22	0.40	1.69±2.8	0.56±0.7	-1.12±2.8	0.22	0.77	0.33±0.7	0.18±0.3	-0.15±0.9	0.69	0.95	0.59	0.76
g_Dehalobacterium	0.55±0.2	0.46±0.2	-0.09±0.3	0.31	0.46	0.53±0.3	0.63±0.5	0.1±0.7	1.00	1.00	0.29±0.2	0.89±0.4	0.6±0.4	0.09	0.61	0.06	0.54
o_YS2_unclass	0.03±0.1	0±0	-0.03±0.1	0.22	0.40	0±0	0±0	0±0	0.44	0.79	0±0	0±0	0±0	0.16	0.61	1.00	1.00
g_Clostridium	0.74±1	0.1±0.2	-0.64±0.9	0.22	0.40	0.31±0.4	0.3±0.3	-0.01±0.5	1.00	1.00	0.4±1	0.26±0.4	-0.14±1.2	0.69	0.95	0.24	0.68
g_Desulfovibrio	0.37±0.2	0.28±0.2	-0.1±0.3	0.16	0.40	0.12±0.1	0.29±0.3	0.16±0.4	0.84	1.00	0.23±0.3	0.45±0.4	0.22±0.6	0.44	0.85	0.24	0.68
f_Ruminococcaceae_1	0.49±0.4	0.2±0.3	-0.29±0.6	0.22	0.40	0.48±0.4	0.35±0.4	-0.13±0.7	0.44	0.79	0.31±0.3	0.19±0.4	-0.12±0.3	0.22	0.70	0.82	0.89
g_Bilophila	0.31±0.3	0.11±0.1	-0.2±0.2	0.16	0.40	0.29±0.3	0.25±0.3	-0.04±0.6	1.00	1.00	0.19±0.2	0.14±0.3	-0.05±0.3	0.06	0.61	0.94	0.96

g_Adlercreutzia	0.07±0.1	0.16±0.1	0.09±0.1	0.06	0.31	0.23±0.1	0.26±0.2	0.03±0.2	1.00	1.00	0.27±0.2	0.25±0.2	-0.02±0.4	0.84	0.95	0.24	0.68
g_Parabacteroides	0±0	0.37±0.4	0.37±0.4	0.06	0.31	0.03±0.1	0.05±0.1	0.02±0.1	1.00	1.00	0±0	0.46±0.2	0.46±0.2	0.03	0.61	0.31	0.68
g_Anaerotruncus	0.16±0.2	0.27±0.3	0.11±0.4	1.00	1.00	0.08±0.1	0.25±0.4	0.17±0.5	0.56	0.79	0.14±0.2	0.16±0.2	0.01±0.4	0.69	0.95	0.48	0.76
g_Coprococcus	0.3±0.3	0±0	-0.3±0.3	0.03	0.27	0.31±0.3	0.09±0.1	-0.22±0.4	0.22	0.77	0.18±0.1	0.17±0.1	0±0.1	0.16	0.61	0.002	**0.08***
f_F16	0.18±0.2	0.05±0.1	-0.13±0.2	0.31	0.46	0.12±0.1	0.23±0.2	0.11±0.2	0.03	0.36	0.24±0.2	0.18±0.2	-0.06±0.3	0.84	0.95	0.59	0.76
f_Enterobacteriaceae	0±0	1.33±3.3	1.33±3.3	0.69	0.83	0±0	0.01±0	0.01±0	0.06	0.36	0±0	0.07±0.2	0.07±0.2	0.84	0.95	0.59	0.76
g_Ruminococcus	0.25±0.3	0.11±0.2	-0.14±0.3	0.16	0.40	0.29±0.2	0.06±0.1	-0.23±0.3	0.06	0.36	0.1±0.2	0.08±0.1	-0.02±0.3	0.69	0.95	0.48	0.76
o_RF32_unclass	0.05±0.1	0.19±0.2	0.14±0.2	0.44	0.57	0±0	0.09±0.1	0.09±0.1	0.06	0.36	0.19±0.4	0.14±0.2	-0.05±0.5	1.00	1.00	0.59	0.76
g_Turicibacter	0±0	0±0	0±0	0.84	0.89	0±0	0.14±0.3	0.14±0.3	0.44	0.79	0±0	0±0	0±0	0.16	0.61	0.39	0.76
g_Butyricicoccus	0.01±0	0.06±0.1	0.04±0.1	0.84	0.89	0.03±0.1	0.12±0.3	0.09±0.3	0.69	0.89	0.11±0.2	0.22±0.5	0.11±0.6	0.44	0.85	0.59	0.76
f_Ruminococcaceae_2	0.09±0.1	0.06±0.1	-0.03±0.1	0.56	0.70	0.01±0	0.02±0	0.01±0	0.31	0.79	0.05±0.1	0.06±0.1	0.01±0.1	0.44	0.85	0.31	0.68
g_Allobaculum	0±0	0.03±0.1	0.03±0.1	0.44	0.57	0±0	0.18±0.4	0.18±0.4	0.44	0.79	0±0	0.01±0	0.01±0	0.84	0.95	0.31	0.68

While majority of ASVs were classified at the genus level, some could only be classified to family (f_), order (o_), or class (c_) levels. Statistical significance of within-group shifts between times was determined by paired Wilcoxon test, while between-group differences in shift (Δ Week 12 – Baseline) were determined by Kruskal-Wallis with Dunn's test and corrected by FDR. The centered log ratio transformed counts of taxa were used in the statiatistcal models. * $q < 0.15$. Data were reported as mean ± SD.

Table S3. Fecal bacterial relative abundance changes in female IL-10$^{-/-}$ mice with isomaltodextrin (IMD) intervention

Taxa	Control					Low Dose IMD					High Dose IMD					Between Group Shift *p* values	FDR
	Baseline	Week 12	Absolute Shift	Within Group *p* values	FDR	Baseline	Week 12	Absolute Shift	Within Group *p* values	FDR	Baseline	Week 12	Absolute Shift	Within Group *p* values	FDR		
**Phylum**																	
F/B Ratio	0.63±0.2	0.59±0.5	-0.04±0.6	0.56	0.88	0.87±0.9	0.63±0.3	-0.24±1	1.00	1.00	0.52±0.4	0.49±0.2	-0.03±0.5	1.00	1.00	0.70	0.88
Bacteroidetes	61.01±6.9	59.62±12.7	-1.4±18.7	1.00	1.00	57.83±19	58.64±9.2	0.81±23.2	0.69	1.00	68.45±18.7	57.64±12.3	-10.8±29.2	0.56	1.00	0.83	0.88
Firmicutes	37.29±6.3	30.86±17.1	-6.4±21.8	0.56	0.88	36.89±21.2	35.17±11.4	-1.7±25.4	1.00	1.00	29.36±17.7	27.93±10.1	-1.43±20.7	0.69	1.00	0.75	0.88
Verrucomicrobia	0±0	3.05±7.5	3.05±7.5	1.00	1.00	2.78±6.8	3.07±7.5	0.29±11.1	0.79	1.00	0±0	11.17±17.6	11.17±17.6	0.37	0.83	0.67	0.88
Proteobacteria	1.03±0.9	1.71±1	0.68±1.3	0.56	0.88	1.93±1.7	1.63±1.4	-0.3±1.9	1.00	1.00	0.78±0.6	2.14±0.8	1.37±1.2	0.03	0.14	0.22	0.87
Tenericutes	0.45±0.5	0.46±0.5	0.01±0.5	1.00	1.00	0.27±0.4	0.43±0.4	0.17±0.5	0.56	1.00	1±1.7	0.52±0.5	-0.48±2	1.00	1.00	0.88	0.88
Cyanobacteria	0±0	3.93±8.2	3.93±8.2	0.37	0.88	0±0	0.62±0.7	0.62±0.7	0.10	0.90	0.14±0.3	0.25±0.6	0.12±0.3	1.00	1.00	0.33	0.87
Actinobacteria	0.13±0.1	0.26±0.2	0.13±0.1	0.06	0.56	0.19±0.2	0.27±0.3	0.09±0.4	0.84	1.00	0.09±0.1	0.31±0.3	0.21±0.3	0.18	0.53	0.78	0.88
TM7	0.1±0.2	0.1±0.1	0.01±0.1	0.58	0.88	0.12±0.1	0.17±0.2	0.05±0.2	0.59	1.00	0.18±0.2	0.04±0.1	-0.15±0.1	0.03	0.14	0.13	0.87
**Genus**																	
f_S24-7	43.87±5.5	34.76±8.8	-9.1±13.4	0.16	0.36	40±15.7	35.32±9.6	-4.6±19.4	0.22	0.70	43.97±11.9	31.39±12.6	-12.57±20	0.56	0.79	0.35	0.66
f_Lachnospiracea_1	19.59±6.7	19.3±10.9	-0.3±17.3	0.31	0.61	19.35±12.4	17.28±7.7	-2.1±16.5	0.56	1.00	16.07±9.5	15.29±6	-0.77±11.3	0.84	0.95	0.35	0.66
g_[Prevotella]	4.89±3.3	10.05±6.3	5.16±6.1	0.56	0.86	4.14±2.4	5.51±4	1.36±4.5	1.00	1.00	12.97±10.8	6.24±4.3	-6.73±13.4	0.56	0.79	0.02	0.38
o_Clostridiales_1	5.26±2.5	2.52±2.3	-2.74±4	0.03	0.24	5.22±3.4	7.77±3.4	2.55±4.9	0.69	1.00	4.06±3.7	6.85±3.3	2.79±5.3	0.22	0.64	0.78	0.83
g_Prevotella	2.89±2	5.46±4.2	2.56±3.4	0.69	0.86	4.93±5.7	8.19±2.6	3.26±6	0.31	0.78	3.2±3.7	4.28±3.3	1.07±6.9	0.56	0.79	0.55	0.77
f_Rikenellaceae	4.68±2.4	3.67±1.4	-1.01±3.2	0.44	0.73	4.26±1.8	5.22±2.9	0.96±4.2	0.84	1.00	4.21±2.4	8.25±11.8	4.04±11.6	1.00	1.00	0.17	0.45
g_Bacteroides	1.4±0.8	2.55±0.9	1.15±1.2	0.31	0.61	1.8±1.3	3.03±2.5	1.23±2.9	0.84	1.00	1.68±1.2	5.41±4.2	3.73±3.6	0.06	0.47	0.10	0.41
g_Oscillospira	2.82±1.4	1.24±0.9	-1.58±2.2	0.06	0.24	2.9±2.3	2.04±0.5	-0.87±2.6	0.69	1.00	1.85±2.3	1.38±0.8	-0.48±2.7	0.69	0.86	0.52	0.77
g_Akkermansia	0±0	3.05±7.5	3.05±7.5	0.69	0.86	2.78±6.8	3.07±7.5	0.29±11.1	0.84	1.00	0±0	11.17±17.6	11.17±17.6	0.31	0.64	0.12	0.41
f_Lachnospiraceae_2	2.66±0.6	1.45±1.2	-1.22±0.9	0.03	0.24	2.53±2	1.71±0.5	-0.82±1.7	0.44	0.96	1.93±1.5	0.77±0.6	-1.16±1.3	0.09	0.47	0.98	0.98
g_Lactobacillus	2.66±3.1	3.06±5.3	0.4±7	0.69	0.86	1.21±1.3	1.92±2.5	0.71±2.4	1.00	1.00	1.08±1.6	1.06±1.2	-0.02±2.2	0.69	0.86	0.12	0.41
o_Clostridiales_2	1.4±1.1	1±1.5	-0.4±2	0.06	0.24	1.92±1.4	2.12±1.7	0.2±2.4	0.84	1.00	1.84±1.4	0.52±0.4	-1.33±1.7	0.06	0.47	0.59	0.77
g_AF12	3.24±1.9	1.58±0.9	-1.66±2	0.09	0.27	2.15±1.3	0.93±0.5	-1.22±1.2	0.22	0.70	1.61±1.3	1.59±2.9	-0.03±3.9	0.56	0.79	0.09	0.41
g_[Ruminococcus]	1.59±0.6	0.87±0.6	-0.72±0.9	0.06	0.24	1.93±1.2	0.68±0.5	-1.26±1.1	0.03	0.27	1.1±0.5	0.19±0.3	-0.91±0.6	0.03	0.47	0.07	0.41
g_Sutterella	0.2±0.2	1.29±0.9	1.09±0.9	0.06	0.24	0.75±1.2	1.02±1.2	0.27±1.6	0.84	1.00	0.29±0.4	1.2±0.8	0.91±0.9	0.09	0.47	0.41	0.70
g_Anaeroplasma	0.45±0.5	0.46±0.5	0.01±0.5	0.56	0.86	0.27±0.4	0.43±0.4	0.17±0.5	0.69	1.00	1±1.7	0.52±0.5	-0.48±2	1.00	1.00	0.12	0.41
g_Dehalobacterium	0.41±0.1	0.31±0.2	-0.1±0.2	0.03	0.24	0.43±0.5	0.7±0.2	0.27±0.5	0.22	0.70	0.33±0.2	0.45±0.2	0.12±0.3	0.31	0.64	0.37	0.67
o_YS2_unclass	0±0	3.93±8.2	3.93±8.2	1.00	1.00	0±0	0.62±0.7	0.62±0.7	0.16	0.68	0.14±0.3	0.25±0.6	0.12±0.3	0.69	0.86	0.26	0.59
g_Clostridium	0.21±0.3	0.11±0.2	-0.09±0.3	0.44	0.73	0.41±0.5	0.16±0.3	-0.25±0.3	0.03	0.27	0.26±0.5	0.25±0.4	-0.02±0.5	1.00	1.00	0.24	0.57
g_Desulfovibrio	0.5±0.4	0.19±0.2	-0.31±0.5	0.09	0.27	0.54±0.5	0.27±0.3	-0.27±0.6	0.31	0.78	0.17±0.2	0.37±0.6	0.21±0.7	0.56	0.79	0.14	0.44
f_Ruminococcaceae_1	0.19±0.3	0.35±0.4	0.16±0.4	0.69	0.86	0.32±0.2	0.23±0.3	-0.09±0.3	0.16	0.68	0.2±0.2	0.13±0.3	-0.07±0.3	0.16	0.64	0.16	0.45
g_Bilophila	0.33±0.4	0.08±0.1	-0.26±0.4	0.09	0.27	0.41±0.3	0.18±0.1	-0.24±0.4	0.31	0.78	0.2±0.2	0.41±0.5	0.21±0.6	0.56	0.79	0.02	0.38
o_Bacteroidales	0.03±0.1	1.49±2.7	1.46±2.7	0.06	0.24	0.2±0.2	0.09±0.1	-0.11±0.3	0.69	1.00	0.71±0.9	0.17±0.3	-0.54±1.1	0.22	0.64	0.95	0.98

g_Adlercreutzia	0.13±0.1	0.26±0.2	0.13±0.1	0.16	0.36	0.19±0.2	0.27±0.3	0.09±0.4	1.00	1.00	0.09±0.1	0.31±0.3	0.21±0.3	0.56	0.79	0.46	0.73
g_Parabacteroides	0±0	0.07±0.2	0.07±0.2	1.00	1.00	0.34±0.8	0.36±0.4	0.01±1	0.44	0.96	0.1±0.2	0.32±0.3	0.22±0.2	0.22	0.64	0.74	0.81
g_Anaerotruncus	0.1±0.2	0.21±0.2	0.11±0.3	0.44	0.73	0.17±0.2	0.25±0.2	0.08±0.3	1.00	1.00	0.04±0.1	0.22±0.4	0.17±0.4	0.31	0.64	0.08	0.41
g_Coprococcus	0.32±0.2	0.06±0.1	-0.26±0.1	0.03	0.24	0.27±0.2	0.14±0.2	-0.13±0.1	0.03	0.27	0.26±0.2	0.1±0.1	-0.17±0.2	0.09	0.47	0.06	0.41
f_F16	0.1±0.2	0.1±0.1	0.01±0.1	1.00	1.00	0.12±0.1	0.17±0.2	0.05±0.2	1.00	1.00	0.18±0.2	0.04±0.1	-0.15±0.1	0.03	0.47	0.65	0.81
f_Enterobacteriaceae	0±0	0.01±0	0.01±0	0.84	0.98	0±0	0±0	0±0	0.09	0.55	0±0	0±0	0±0	0.84	0.95	0.69	0.81
g_Ruminococcus	0.06±0.1	0.11±0.1	0.04±0.2	0.84	0.98	0.08±0.1	0.04±0.1	-0.03±0.2	0.84	1.00	0.06±0.1	0.18±0.4	0.12±0.4	1.00	1.00	0.69	0.81
o_RF32_unclass	0±0	0.15±0.2	0.15±0.2	0.16	0.36	0.22±0.5	0.16±0.3	-0.07±0.7	1.00	1.00	0.12±0.2	0.16±0.2	0.04±0.2	0.31	0.64	0.57	0.77
g_Turicibacter	0±0	0.17±0.4	0.17±0.4	1.00	1.00	0±0	0.04±0.1	0.04±0.1	0.69	1.00	0±0	0.52±1	0.52±1	0.31	0.64	0.74	0.81
g_Butyricicoccus	0±0	0.03±0.1	0.03±0.1	0.69	0.86	0.08±0.2	0±0	-0.08±0.2	0.03	0.27	0.18±0.3	0.02±0	-0.16±0.4	0.44	0.79	0.31	0.66
f_Ruminococcaceae_2	0±0	0.07±0.2	0.07±0.2	1.00	1.00	0.06±0.1	0.09±0.1	0.03±0.1	1.00	1.00	0.08±0.1	0.01±0	-0.07±0.1	0.22	0.64	0.47	0.73
g_Allobaculum	0±0	0±0	0±0	0.31	0.61	0±0	0±0	0±0	0.09	0.55	0±0	0±0	0±0	0.84	0.95	0.35	0.66

While majority of ASVs were classified at the genus level, some could only be classified to family (f_), order (o_), or class (c_) levels. Statistical significance of within-group shifts between times was determined by paired Wilcoxon test, while between-group differences in shift (Δ Week 12 – Baseline) were determined by Kruskal-Wallis with Dunn's test and corrected by FDR. The centered log ratio transformed counts of taxa were used in the statiatistcal models. * $q < 0.15$. Data were reported as mean ± SD.

Table S1. Histology scoring assessment in male and female IL-10$^{-/-}$ mice with isomaltodextrin (IMD) for 12 weeks

	Male				Female			
	Control	LD	HD	*p*	Control	LD	HD	*p*
Histology score	2.50±2.35	2.00±2.53	2.50±2.26	0.88	2.00±1.90	2.00±2.76	3.50±1.64	0.43
Neutrophil infiltration score	1.00±0.89	0.67±1.03	0.83±0.98	0.74	1.00±0.89	0.67±0.82	1.33±0.82	0.35
Lymphocyte infiltration score	1.17±0.98	0.83±0.98	1.33±1.03	0.47	0.83±0.75	0.83±0.98	1.67±0.82	0.90
Tissue injury score	0.33±0.82	0.50±0.84	0.33±0.52	0.94	0.17±0.41	0.50±1.22	0.50±0.55	0.45

Statistical comparisons between-groups were by ANCOVA with permutations (n=999), adjusted by the food consumption AUC. Post hoc multiple comparison used Tukey's test. Data were reported as mean ± SD.
